# Facteurs associés à la vaccination contre le virus du papillome humain dans un contexte de passage à l´échelle au Sénégal: enquête cas-témoins auprès des parents

**DOI:** 10.11604/pamj.2021.39.137.29229

**Published:** 2021-06-17

**Authors:** Mbouna Ndiaye, Bernard Sawadogo, Ibrahima Sonko, Ibrahima Oumar Ba, Mamadou Makhtar Mbacke Leye

**Affiliations:** 1 Ministère de la Santé, Dakar, Sénégal,; 2 Réseau Africain d´Épidémiologie de Terrain (AFENET), Kampala, Ouganda,; 3 Centre des Opérations d´Urgence Sanitaire (COUS), Dakar, Sénégal,; 4 Organisation Mondiale de la Santé, Dakar, Sénégal,; 5 Service de Médecine Préventive et Santé Publique/Université Cheikh Anta Diop de Dakar, Dakar, Sénégal

**Keywords:** Vaccination, virus, papillome humain, facteurs, Sénégal, Vaccination, human papilloma virus, factors, Senegal

## Abstract

**Introduction:**

après une phase pilote, le Sénégal est le premier pays en Afrique de l´Ouest à introduire le vaccin contre le cancer du col de l´utérus dans le Programme élargi de vaccination. Malgré la gratuité et la disponibilité du vaccin, la couverture est restée faible. L´objectif de cette étude était d´identifier les facteurs associés à la vaccination des filles contre le virus du papillome humain.

**Méthodes:**

il s´agissait d´une étude analytique de type cas-témoin réalisée du 4 au 20 janvier 2020 à Dakar. La population d´étude était constituée de parents ou tuteurs de filles âgées de 9 à 10 ans. Nous avons réalisé un échantillonnage en grappes, des entretiens structurés directs et une revue documentaire. Les caractéristiques sociodémographiques, les connaissances des parents/tuteurs et les informations sur l´acte vaccinal ont été collectées à l´aide d´un questionnaire standardisé. Une régression logistique a permis d´estimer les odds-ratio.

**Résultats:**

au cours de cette étude, 510 cas et 510 témoins, soient 1020 parents/tuteurs étaient interviewés. Les facteurs significatifs associés à la vaccination des filles étaient: l´instruction des parents/tuteurs (ORa=1,97; [1,81-2,25]), la connaissance de la maladie (ORa=3,05; [2,75-4,53], le revenu élevé du ménage (ORa=1,21; [1,13-1,85]), la crainte des effets secondaires (ORa=0,35; [0,27-0,44]), la réception de messages via internet/réseaux sociaux (ORa=0,54; [0,41-0,92]) et les horaires de vaccination adaptées à la communauté (ORa= 2,12 [1,59-2,64]).

**Conclusion:**

la vaccination des filles peut être améliorée par un renforcement des connaissances des parents à travers des canaux appropriés et une meilleure organisation des services de santé.

## Abstract (en)

**Introduction:**

after a pilot phase, Senegal is the first country in West Africa to introduce cervical cancer vaccine into its Expanded Program on Immunization. Despite the gratuity and availability of the vaccination, coverage was low. The purpose of this study was to identify factors associated with HPV vaccination coverage in girls .

**Methods:**

we conducted a case-control analytical study from 4^th^ to 20^th^ January 2020 in Dakar. The study population consisted of parents or guardians of girls aged 9 to 10. We performed cluster sampling, direct structured interviews and a literature review. Socio-demographic features, parents/guardians’ knowledges and information about vaccination procedure were collected using a standardized questionnaire. Logistic regression was used to estimate the odds ratio.

**Results:**

during this study, 510 cases and 510 controls and 1020 parents/guardians were interviewed. Significant factors associated with vaccination of girls were: parents/guardians’ education (OR=1,97; [1,81-2,25]), knowledge of the disease (OR=3,05; [2,75-4,53], high household income (OR=1,21; [1,13-1,85]), fear of side effects (OR=0,35;[ 0,27-0,44]), reception of messages via internet/social networks (OR=0,54; [0,41-0,92]) and vaccination schedules for the community (OR= 2,12 [1,59-2,64]).

**Conclusion:**

vaccination of girls can be improved by strengthening parents’ knowledge through appropriate channels and a better organization of health services.

## Introduction

Le cancer du col de l´utérus constitue un problème majeur de santé publique dans le monde avec une incidence supérieure à 530 000 par an et une mortalité de près de 275 000 décès par an [1]. Environ 90% des décès surviennent dans les pays en voie de développement où les taux d´incidence sont plus élevés [2]. L´Organisation mondiale de la santé (OMS) recommande la vaccination des filles âgées de 9 à 13 ans contre le virus du papillome humain (VPH) comme mesure de santé publique la plus rentable contre le cancer du col utérin [3]. Des projets de démonstration sur la vaccination contre le VPH ont permis d´obtenir des résultats probants en Inde, au Pérou, en Ouganda et au Vietnam avec des couvertures allant de 77,2 et 96,1% [4]. En revanche, dans d´autres pays comme la France, le taux de couverture vaccinale était inférieur à 30% [5]. En effet, dans les pays où la vaccination est effective, elle s´est heurtée parfois aux craintes sur l´innocuité du vaccin [5,6] au manque de motivation des parents et à la méconnaissance de la maladie [7].

Au Sénégal, le cancer du col de l´utérus est le premier cancer gynécologique. Le nombre de nouveaux cas annuels est estimé à 1500 soit 34% de tous les cas annuels de cancer. Il s´agit de la première cause de décès dus au cancer chez les femmes âgées de 15 à 44 ans [8]. Après une phase pilote, le Sénégal est le premier pays en Afrique de l´Ouest à introduire le vaccin contre le cancer du col de l´utérus le 31 octobre 2018 dans son Programme élargi de vaccination (PEV). Dans le processus de mise en œuvre, l´option d´une collaboration multisectorielle à tous les niveaux en particulier le secteur de l´éducation (formelle et religieuse) a été choisie. Les écoles et autres institutions scolaires sont considérées comme des sites occasionnels de vaccination.

Malgré la gratuité et la disponibilité du vaccin dans le programme, la couverture vaccinale est restée faible dans la région de Dakar. En 2019, seul 1/3 des filles de 9-10 ans étaient complètement vaccinées [9]. Les facteurs associés à la vaccination des filles contre le VPH ne sont pas connus. Dans le continent africain, en raison de la non-introduction du vaccin dans la plupart des programmes nationaux, la question est très peu étudiée. Les quelques rares recherches déjà menées ont exploré le sujet dans un contexte expérimental (avant l´introduction du vaccin dans le PEV) [10]. En Afrique de l´Ouest, aucune étude à notre connaissance sur la vaccination contre le virus du papillome humain dans un contexte de routine (Programme Elargi de vaccination) n´a encore été rapportée. Notre étude s´inscrit dans le cadre d´une évaluation post-introduction du vaccin comme le recommande l´OMS [11], afin de repérer les lacunes programmatiques et d´apporter des améliorations au programme de vaccination. Cette étude avait pour objectif d´identifier les facteurs associés à la vaccination des jeunes filles de 9 à 13 ans contre le VPH.

## Méthodes

**Cadre d´étude**: situé à l´extrême Ouest du continent africain, la région de Dakar abrite 23,2% de la population du Sénégal sur une superficie estimée à 0,28% du territoire national [12]. La quasi-totalité de la cible vaccinale (99%) était atteinte en stratégie fixe et 1% en stratégie avancée. En dépit de cette apparente facilité, il existait des zones défavorisées ou d´accès difficile particulièrement dans les communes de Keur Massar et de Malika où les objectifs de couvertures vaccinales anti-VPH n´étaient pas atteints. La cible officielle de la vaccination anti-VPH dans ces 2 communes, objet de notre étude était estimée à 10 774 filles. La couverture vaccinale était de 47,3% en 2019. Au cœur de la banlieue dakaroise, les 2 communes disposaient de 13 unités de vaccination réparties dans 9 aires de santé. Dans le domaine des équipements, 87% des unités de vaccination possédaient une chaine de froid homologuée [9].

**Type et période de l´étude**: il s´agissait d´une étude analytique de type cas-témoin conduite du 4 au 20 janvier 2020 à Dakar.

**Population d´étude**: la population d´étude était constituée de parents ou tuteurs de filles âgées de 9 à 10 ans. Un cas était défini comme un parent ou tuteur dont la fille âgée de 9 à 10 ans avait reçu deux doses de vaccin anti VPH notifiées sur la carte de vaccination. Un témoin était défini comme un parent ou tuteur vivant dans la même concession ou dans le voisinage du cas, dont la fille âgée de 9 à 10 ans n´avait pas été vaccinée contre le VPH.

**Echantillonnage**: la taille de l´échantillon a été calculée avec le logiciel Open Epi version 3 selon le principe d´une étude cas témoins non apparié [13]. Pour un niveau de confiance bilatérale de 95%, une puissance de 80%, un ratio cas/témoins de 1, une valeur minimale de 1,8 pour l´odds-ratio et un pourcentage hypothétique de témoins exposés de 23% [5,11,14], la taille minimale théorique de l´échantillon était de 241 cas et 241 témoins selon la méthode de Fleiss avec correction de continuité. En tenant compte d´éventuels non répondants à l´étude (5%), la taille est égale à (2 x 241) + 5%, soit 506 cas et 506 témoins. Nous avons réalisé un échantillonnage en grappes à plusieurs degrés: a) échantillonnage des aires de santé: il a été exhaustif; nous avons établi la liste des 9 aires de santé avec les populations de filles (9-10 mois) cibles de la vaccination [9]; b) sélection des grappes: chaque quartier de l´aire de santé était considéré comme une grappe. Pour le choix des 30 grappes, nous avons réalisé un sondage systématique connaissant l´effectif cumulé de la population de filles ciblées (10774); c) choix des concessions: l´enquêteur a utilisé une carte pour déterminer le centre à partir duquel une direction a été choisie au hasard. En cas de non disponibilité d´une carte, l´enquêteur s´est positionné dans un lieu public. La première concession se trouvant sur cette direction a été enquêtée; d) sélection des unités statistiques: une fois au domicile et après avoir identifié un cas, nous avons choisi dans la concession un témoin, à défaut dans la concession voisine.

### Variables de l´étude

**Variable dépendante**: représentée par le statut vaccinal VPH des filles âgées de 9 à 10 ans.

**Variables indépendantes**: âge, sexe, situation matrimoniale, profession, niveau d´instruction, revenus du ménage, connaissance de la maladie, facteurs de vaccination liés aux connaissances, à l´information, à la motivation, à l´offre et à l´organisation des services de vaccination.

**Collecte des données**: des entretiens structurés direct (face à face) à l´aide d´un questionnaire d´enquête standardisé ont été réalisés. Une revue des cartes de vaccination a aussi été réalisée. La phase de collecte de données a été précédée d´une enquête pilote pour tester les outils au niveau d´une zone non concernée par l´étude.

**Gestion et analyse des données**: les données ont été saisies et analysées à l´aide du logiciel Epi info version 7.2.4. Les variables qualitatives ont été synthétisées par leurs fréquences et celles quantitatives par leurs moyennes encadrées par l´écart-type. Le test de Chi-deux ou Exact de Fisher ont été utilisés selon leurs conditions d´application pour comparer les proportions. Une analyse univariée a permis de retenir les facteurs associés au seuil de p <0,20. Une régression logistique a été utilisée pour identifier les facteurs associés à la vaccination anti-VPH. Une démarche pas à pas descendante a permis de retenir les variables associées à la vaccination au seuil p < 5%. L´adéquation du modèle final a été vérifiée par le test d´Hosmer-Lemeshow.

**Considérations éthiques**: le protocole de l´étude a été soumis aux autorités de la santé pour approbation. Une lettre d´information a été adressée au médecin chef et aux responsables de zone. La participation à cette étude était volontaire et seuls les participants ayant donné leur consentement libre et éclairé étaient inclus dans l´étude. Les questionnaires étaient anonymisés et les interviews ont été réalisées en respectant la confidentialité.

## Résultats

**Description des cas et des témoins**: au cours de cette étude, 510 cas et 510 témoins, soient 1020 parents/tuteurs ont été interviewés. La moyenne d´âge était de 46,0 ± 8,2 ans chez les parents/tuteurs dont les filles étaient vaccinées et 44,5 ± 5,0 ans chez ceux dont les filles n´étaient pas vaccinées. Parmi les parents/tuteurs des filles vaccinées, 44,3% n´étaient pas instruits contre 64,1% chez ceux dont les filles étaient non vaccinées. Les ménages qui avaient un revenu mensuel inférieur à 110 000 FCFA étaient de 71,2% chez les cas et de 81,6% parmi les témoins. Les parents/tuteurs des filles vaccinées n´avaient signalé aucune manifestation adverse post-immunisation (MAPI) dans 64,7% des cas. Les MAPI enregistrées étaient: la douleur (17,8%), le gonflement (10,8%) et la rougeur locale (8,2%). Aucune réaction grave n´a été observée. Concernant la décision vaccinale des parents/tuteurs des filles non vaccinées 49, 4% hésitaient à leur faire bénéficier de l´acte vaccinal et 32,2% manifestaient un refus (Tableau 1). Les principales sources d´informations des parents étaient: les relais communautaires, la télévision/radio puis l´école. La proportion de parents/tuteurs qui s´informaient à partir des relais communautaires était de 82,4% chez les cas et 66,7% parmi les témoins. Les parents/tuteurs qui avaient cité la télévision et la radio étaient de 84,7% chez les cas et de 80,6% chez les témoins. Le milieu scolaire, comme source d´information des parents, représentait 61,2% (chez les cas) et 57,8% (parmi les témoins).

**Tableau 1:** répartition selon la décision vaccinale des parents/tuteurs, Dakar, 2018-2020

Décision des parents/tuteurs	Cas n(%)	Témoins n(%)
Accord	510(100,0)	94(18,4)
Hésitation	-	252(49,4)
Refus	-	164(32,2)
**Total**	510(100,0)	510(100,0)

**Analyse univariée**: l´instruction des parents/tuteurs influençait la vaccination des filles. Le sexe n´était pas associé significativement à la vaccination (Tableau 2). L´accueil satisfaisant favorisait la vaccination (Tableau 3).

**Tableau 2:** caractéristiques sociodémographiques des parents associées à la vaccination anti-VPH en analyse univariée des filles de 9 à 10 ans, Dakar, 2018-2020

Caractéristiques sociodémographiques		Cas n(%)	Témoins n(%)	OR IC 95%	p
Tranche d´âge	≥35	464 (91,0)	455 (89,2)	1,22 [0,81-1,84]	0,348
(an)	< 35	46 (9,0)	55 (10,8)	1	
Sexe	Féminin	397 (77,8)	382 (74,9)	1,18 [0,88-1,57]	0,270
	Masculin	113 (22,2)	128 (25,1)	1	
Situation matrimoniale	En union	473 (92,7)	465 (91,2)	1,24 [0,79-1,95]	0,360
	Non en union	37 (7,3)	45 (8,8)	1	
Profession	Avec	416 (81,6)	409 (80,2)	1,09 [0, 80-1,49]	0,578
	Sans	94 (18,4)	101 (19,8)	1	
Instruction	instruit	284 (55,7)	183 (35,9)	2,24 [1,75-2,89]	0.000
	Non instruit	226 (44,3)	327 (64,1)	1	
Revenu mensuel du ménage (FCFA)	≥ 110 000	147 (28,8)	94 (18,4)	1,79 [1,34-2.41]	0.000
	< 110 000	363 (71,2)	416 (81,6)	1	

**Tableau 3:** autres facteurs associés à la vaccination anti-VPH en analyse univariée des filles de 9 à 10 ans, Dakar, 2018-2020

Facteurs		Cas n(%)	Témoins n(%)	ORa IC 95%	p
**Facteurs liés aux connaissances**					
Maladie ciblée (cancer du col)	Oui	407 (79,8)	221 (43,3)	5,16 [3,91-6,82]	0,000
	Non	103 (20,2)	289 (56,7)	1	
Gratuité du vaccin	Oui	464 (91,0)	455 (89,2)	1,22 [0,81-1,84]	0,348
	Non	46 (9,0)	55 (10,8)	1	
**Facteurs liés à l´information**					
Crainte des effets secondaires du vaccin	Oui	193 (37,8)	381 (74,7)	0,21 [0,16-0,27]	0,000
	Non	317 (62,2)	129 (25,3)	1	
Leaders communautaires	Oui	307 (60,2)	193 (37,8)	2,48 [1,93-3,20]	0,000
	Non	203 (39,8)	317 (62,2)	1	
Internet/réseaux sociaux	Oui	125 (24,5)	312 (61,2)	0,21 [0,16-0,27]	0,000
	Non	385 (75,5)	198 (38,8)	1	
**Facteurs liés à la motivation**					
Disponibilité des parents	Oui	119 (23,3)	141 (27,6)	0,79 [0,60-1,06]	0,114
	Non	391 (76,7)	369 (72,4)	1	
Négligence des Parents	Oui	432 (84,7)	411 (80,6)	1,33 [0,96-1,85]	0,083
	Non	78 (15,3)	99 (19,4)	1	
**Facteurs liés à l´offre et à l´organisation des services de vaccination**					
Conditions d´accueil satisfaisant	Oui	424 (83,1)	340 (66,7)	2,46 [1,83-3,31]	0,000
	Non	86 (16,9)	170 (33,3)	1	
Horaire de vaccination adaptée	Oui	190 (37,3)	98 (19,2)	2,49 [1,88-3,32]	0,000
	Non	320 (62,7)	412 (80,8)	1	
Lieu de vaccination	Ecole	312 (61,2)	295 (57,8)	1,15 [0,89-1,47]	0,279
	Ailleurs	198 (38,8)	215 (42,2)	1	

**Analyse multivariée**: la connaissance de la maladie par les parents/tuteurs était associée à la vaccination. Les horaires des séances adaptées à la communauté favorisaient la vaccination (Tableau 4).

**Tableau 4:** facteurs associés à la vaccination anti-VPH en analyse multivariée des filles de 9 à 10 ans, Dakar, 2018-2020

Facteurs		Cas n(%)	Témoins n(%)	ORa IC 95%	p
Instruction	Instruit	284 (55,7)	183 (35,9)	1,97 [1,81-2,25]	**0,010**
	Non instruit	226 (44,3)	327 (64,1)	1	
Revenus mensuels du ménage (FCFA)	≥ 55 000	147 (28,8)	94 (18,4)	1,21 [1,13-1,85]	**0,000**
	< 55 000	363 (71,2)	416 (81,6)	1	
Connaissance maladie ciblée	Oui	407 (79,8)	221 (43,3)	3,05 [2,75-4,53]	**0,000**
	Non	103 (20,2)	289 (56,7)	1	
Crainte effets secondaires	Oui	193 (37,8)	381 (74,7)	0,35 [0,27-0,44]	**0,000**
	Non	317 (62,2)	129 (25,3)	1	
Leaders communautaires	Oui	307 (60,2)	193 (37,8)	1,10 [0,86-2,16]	0,492
	Non	203 (39,8)	317 (62,2)	1	
Internet/Réseaux sociaux	Oui	125 (24,5)	312 (61,2)	0,54 [0,41-0,92]	**0,001**
	Non	385 (75,5)	198 (38,8)	1	
Disponibilité des parents	Oui	119 (23,3)	141 (27,6)	0,30 [0,23-1,97]	0,080
	Non	391 (76,7)	369 (72,4)	1	
Négligence des Parents	Oui	432 (84,7)	411 (80,6)	1,05 [0,82-1,71]	0,067
	Non	78 (15,3)	99 (19,4)	1	
Accueil satisfaisant	Oui	424 (83,1)	340 (66,7)	2,08 [0,72-2,45]	0,130
	Non	86 (16,9)	170 (33,3)	1	
Horaire de vaccination adaptée	Oui	190 (37,3)	98 (19,2)	2,12 [1,59-2,64]	**0,000**
	Non	320 (62,7)	412 (80,8)	1	

## Discussion

Dans notre étude, les facteurs significatifs associés à la vaccination des filles étaient: l´instruction et la connaissance de la maladie par parents/tuteurs, le revenu élevé du ménage, la crainte des effets secondaires, la réception de messages via internet/réseaux sociaux et les horaires de vaccination adaptées à la communauté. Notre étude n´a pas trouvé d´association statistiquement significative dans la relation entre le sexe des parents et la vaccination des filles contre le VPH. Les résultats sont concordants avec ceux de Adama *et al*. [10]. Cette situation montre la position presque similaire des femmes et des hommes vis à vis de la vaccination. Il s´agit d´un progrès remarquable si l´on prend en compte certains stéréotypes sociaux qui considèrent que « la vaccination est une affaire de femmes ». Par contre dans une étude menée au Maroc [15], les parents refusant la vaccination au HPV étaient majoritairement de sexe masculin. Des recherches antérieures ainsi que cette présente étude ont révélé que l´information des parents sur le cancer du col et le VPH est considérablement associée à la vaccination [16,17]. Dans une enquête menée par Naima Baddouh *et al*. [15], le taux d´acceptabilité du vaccin par les parents avait augmenté de 63% à 82% après avoir eu des connaissances sur le cancer du col et le virus du papillome humain. Au Kenya, le manque d´informations sur le vaccin a été identifié comme un obstacle à la vaccination [6]. Ce qui montre qu´accroître la connaissance des parents sur le vaccin peut influencer positivement la décision de vacciner. Il existait un lien entre la pratique vaccinale et le pouvoir économique des ménages. Le rôle des facteurs socio-économiques dans la vaccination a été noté dans plusieurs études en Afrique [6,7,16]. Quoique gratuite dans les programmes nationaux, la vaccination peut avoir des coûts supplémentaires onéreux pour les familles pauvres, il s´agit par exemple des frais de transport. Une autre dimension non monétaire de la pauvreté a été prise en considération dans des recherches [7] qui ont montré que la possession d´articles électroménagers augmentaient la proportion de filles vaccinées de manière significative (ORa=12,71; [2,11-76,75]. Toutefois, la relation vaccination et pauvreté n´est pas toujours aisée à comprendre. En dehors de l´aspect économique, il existe d´autres facteurs qui entrent en jeu.

La peur des effets secondaires était un obstacle à la vaccination. Cette préoccupation majeure a été soulevée par d´autres études [18,19,20]. C´est une question très sérieuse prise en compte dans les programmes nationaux de vaccinations du monde entier car pouvant annihiler les efforts de prévention du cancer du col de l´utérus et compromettre la réussite de la vaccination en générale. Dans notre étude, les effets secondaires étaient rares, essentiellement des réactions locales, par exemple une douleur (17,8%), un gonflement (10,8%). Des recherches antérieures ont aussi conclu à la rareté des effets secondaires et à la nécessité d´une information plus large sur les capacités protectrices du vaccin et sur son innocuité [21,22]. Malheureusement, dans notre enquête, cette innocuité a été entachée de rumeurs sans preuve véhiculées à travers les réseaux sociaux constituant ainsi un frein à la vaccination. Les contre-performances enregistrées dans le cadre de la vaccination anti VPH au niveau de plusieurs pays, reflètent une défiance croissante à l´égard de la vaccination au sein de la population et des professionnels de santé, défiance alimentée par une diffusion dans les réseaux sociaux d´opinions hostiles et non fondées, reprises par certains médias [20,23,24]. Les horaires adaptés à la communauté favorisaient la vaccination. Des auteurs annonçaient déjà que la couverture vaccinale adéquate est tributaire d´une bonne adaptation des horaires des séances. Selon Simon F. *et al*. [25], les heures de vaccination inadaptées étaient significativement associées aux mères d´enfants partiellement vaccinés. Le lieu où l´acte vaccinal était réalisé n´influençait pas la vaccination. Adama *et al*. [10] ont rapporté des résultats similaires à propos de l´endroit où se tenaient les séances de vaccination. Cependant l´école, joue un rôle fondamental dans la stratégie d´implémentation à l´échelle du programme de vaccination contre le VPH. En effet, l´essentiel de la cible 9-10 ans est retrouvé dans le milieu scolaire. Quel que soit l´option de vaccination (école ou ailleurs), elle devra prendre en compte les filles fréquentant les structures d´éducation non formelles, religieuses ou privées et celles non scolarisées se trouvant dans les domiciles.

## Conclusion

La vaccination des filles était associée à l´instruction des parents, au revenu des ménages, à la connaissance de la maladie, à la peur des effets secondaires du vaccin, la réception de messages via internet et à l´adaptation des horaires de vaccination. Le renforcement des connaissances des parents par la sensibilisation fondée sur des messages authentiques à travers des canaux appropriés permettra d´améliorer l´acceptabilité vaccinale. Une meilleure organisation des services de santé est attendue pour la réussite du programme de vaccination.

### Etat des connaissances sur le sujet

- L´acceptabilité de la vaccination en général et particulièrement du vaccin contre le HPV est un problème inquiétant aussi bien dans les pays développés que ceux à ressources limitées;
- Malgré la disponibilité du vaccin anti HPV à Dakar, la couverture vaccinale est restée faible.

### Contribution de notre étude à la connaissance

- Notre étude a confirmé l´hésitation vaccinale vis à vis de l´antigène HPV auprès des parents, une situation qui pourrait compromettre les acquis dans le domaine du PEV;
- Notre étude a pu identifier plusieurs obstacles dans un contexte de passage à l´échelle de la vaccination anti VPH: l´analphabétisme des parents, le faible revenu des ménages, l´insuffisance des connaissances sur le cancer du col de l´utérus, la peur des effets secondaires du vaccin, l´influence négative des rumeurs non fondées (sur le vaccin) émanant des réseaux sociaux et l´inadaptation des horaires de vaccination dans les structures sanitaires;
- Nous avons porté à l´attention des autorités sanitaires sur la nécessité d´améliorer les connaissances des parents et l´organisation de l´offre de service vaccinale.

## Remerciements

Nous adressons nos remerciements aux communautés et aux institutions qui nous ont soutenues dans la réalisation de ce travail: autorités administratives et sanitaires, collectivités territoriales, etc.
